# Expert Opinion on the Management of Growth Hormone Deficiency in Brain Tumor Survivors: Results From an Italian Survey

**DOI:** 10.3389/fendo.2022.920482

**Published:** 2022-07-14

**Authors:** Natascia Di Iorgi, Giovanni Morana, Marco Cappa, Ludovico D’Incerti, Maria Luisa Garrè, Armando Grossi, Lorenzo Iughetti, Patrizia Matarazzo, Maria Parpagnoli, Gabriella Pozzobon, Mariacarolina Salerno, Iacopo Sardi, Malgorzata Gabriela Wasniewska, Stefano Zucchini, Andrea Rossi, Mohamad Maghnie

**Affiliations:** ^1^ Department of Pediatrics, IRCCS Istituto Giannina Gaslini, Endo-European Reference Networks (ERN) Center for Rare Endocrine Conditions, Genova, Italy; ^2^ Department of Neuroscience, Rehabilitation, Ophthalmology, Genetics, Maternal and Child Health (DINOGMI), University of Genova, Genoa, Italy; ^3^ Department of Neurosciences, Neuroradiology Unit, University of Turin, Turin, Italy; ^4^ Unit of Endocrinology, Bambino Gesù Children’s Hospital, Istituti di Ricovero e Cura a Carattere Scientifico (IRCCS), Rome, Italy; ^5^ Department of Pediatric Radiology, Meyer Children’s Hospital, Florence, Italy; ^6^ Neuro-Oncology Unit, IRCCS Istituto Giannina Gaslini, Genova, Italy; ^7^ Unit of Endocrine Pathology of Post-Tumoral and Chronic Diseases, Bambino Gesù Children’s Hospital, Rome, Italy; ^8^ Pediatric Unit, Department of Medical and Surgical Sciences of the Mother, Children and Adults. University of Modena and Reggio Emilia, Modena, Italy; ^9^ Department of Pediatric Endocrinology, Regina Margherita Children’s Hospital, A.O.U. Città della Salute e della Scienza, Turin, Italy; ^10^ Health Sciences Department, Children With Clinical Complex Needs Meyer Children’s Hospital, Florence, Italy; ^11^ Pediatric Unit, IRCCS San Raffaele Scientific Institute, Vita Salute San Raffaele University, Endo-European Reference Networks (ERN) Center for Rare Endocrine Conditions, Milan, Italy; ^12^ Pediatric Unit, Department of Translational Medical Sciences, University Federico II, Naples, Italy; ^13^ Neuro-Oncology Unit, Department of Pediatric Oncology, Meyer Children’s Hospital, Florence, Italy; ^14^ Unit of Paediatrics, Department of Human Pathology of Adulthood and Childhood, University of Messina, Messina, Italy; ^15^ Pediatric Endocrine Unit, IRCCS Azienda Ospedaliero-Universitaria di Bologna, Endo-European Reference Networks (ERN) Center for Rare Endocrine Conditions, Bologna, Italy; ^16^ Neuroradiology Unit, IRCCS Istituto Giannina Gaslini, Genoa, Italy; ^17^ Department of Health Sciences (DISSAL), University of Genoa, Genoa, Italy

**Keywords:** growth hormone deficiency, pediatric brain tumor survivors, brain MRI, radiotherapy, recombinant human growth hormone (rhGH)

## Abstract

**Background:**

Growth hormone deficiency (GHD) is the first and most common endocrine complication in pediatric brain tumor survivors (BTS). GHD can occur due to the presence of the tumor itself, surgery, or cranial radiotherapy (CRT).

**Aims:**

This study aimed to evaluate management and adherence to current guidelines of the Italian centers engaged in the diagnosis and follow-up of GHD patients with BTS.

**Methods:**

A multidisciplinary scientific board of pediatric endocrinologists, oncologists and radiologists with neuroimaging expertise discussed and reviewed the main issues relating to the management of GHD in pediatric BTS and developed a survey. The survey included questions relating to organizational aspects, risk factors, diagnosis, definition of stable disease, and treatment. The online survey was sent to an expanded panel of specialists dedicated to the care of pediatric BTS, distributed among the three specialty areas and throughout the country (23 Italian cities and 37 Centers).

**Results:**

The online questionnaire was completed by 86.5% (32 out of 37) of the Centers involved. Most had experience in treating these patients, reporting that they follow more than 50 BTS patients per year. Responses were analyzed descriptively and aggregated by physician specialty. Overall, the results of the survey showed some important controversies in real life adherence to the current guidelines, with discrepancies between endocrinologists and oncologists in the definition of risk factors, diagnostic work-up, decision-making processes and safety. Furthermore, there was no agreement on the neuroimaging definition of stable oncological disease and how to manage growth hormone therapy in patients with residual tumor and GHD.

**Conclusions:**

The results of the first Italian national survey on the management of GHD in BTS highlighted the difference in management on some important issues. The time to start and stop rhGH treatment represent areas of major uncertainty. The definition of stable disease remains critical and represents a gap in knowledge that must be addressed within the international guidelines in order to increase height and to improve metabolic and quality of life outcomes in cancer survivors with GHD.

## Introduction

Brain tumors are the most common solid pediatric malignancies (1). Owing to improvements in treatment and supportive care, the current 5-year overall survival rate is >80% (2). However, these patients are at risk of long-term morbidities related to the tumor itself or to its treatment, with endocrine dysfunctions being the most common complications (3). Recent data indicate that 40-50% of pediatric BTS will develop at least one endocrinopathy over the course of their lifetime (4, 5). The risk of developing endocrine disorders is dependent on a wide range of variables, including age, gender, length of follow-up, genetic background, primary disease (e.g., histologic diagnosis, tumor location, hydrocephalus), and treatment (e.g., surgery, chemotherapy, radiation, use of checkpoint inhibitors, protocols) (6, 7). Radiation exposure of key endocrine organs (e.g., hypothalamus, pituitary, thyroid, and gonads) is the single most important risk factor and confers to these patients an extremely high risk of developing an endocrine abnormality over time. Cranial radiation therapy (CRT), total body irradiation (TBI), spinal irradiation (CSRT) can variably damage the hypothalamic/pituitary area leading to hypopituitarism (7, 8). Indeed, a recent evidence-based recommendation for surveillance of cancer survivors highlighted high quality evidence of the risk for growth hormone deficiency (GHD) associated with RT exposure of the HP region, moderate for corticotropin deficiency, and low for thyrotropin and gonadotropin deficiency and central precocious puberty (7). A cumulative dose of 16.1 Gy to the hypothalamus is considered to be the mean radiation dose required to achieve a 50% risk of GHD at 5 years (9). Importantly, radiation-induced abnormalities are in general both dose- and time-dependent, such that the higher the dose and the longer the interval following treatment, the greater the risk. Thus, endocrine disorders may not develop for decades after the completion of cancer treatment. Therefore, lifelong surveillance is of critical importance in at risk patients (10–12).

GHD is the earliest and most frequent pituitary disorder, affecting 12.5% of patients after an average follow-up of 6.6 years (11, 13). The risk of developing GHD depends on tumor growth, surgery within or near the hypothalamic-pituitary region, radiation doses, follow-up duration, and other reported factors (7, 12–15). CRT initially affects the hypothalamus, which is more sensitive to irradiation than the anterior pituitary gland (16). In the study of Laughton et al, the cumulative incidence of GHD exceeded 90% at 4 years of follow-up after medulloblastoma treatment (15). Other factors may influence the prevalence of GHD, including the population studied, type of stimulation test, and GH peak after stimulation used to establish a diagnosis (14, 17)

BTS with GHD show decreased growth velocity and may end up with short stature if left untreated. For these reasons, the American Endocrine Society guidelines (18) recommend a prospective evaluation of linear growth in all children who received CRT (every 6-12 months). In cases of growth deceleration, a GH stimulation test should be performed to confirm or exclude GHD; age-adjusted IGF-I levels may be useful in screening for severe GHD but have limited utility when using a -2 SD cut-off (19–21). GH provocative testing should be performed in all patients with growth deceleration, but testing is not necessary in patients with three or more pituitary deficiencies (18). Moreover, it is suggested that the same stimulation tests be used as in the general population, including insulin tolerance test (ITT), glucagon, arginine, levodopa, and clonidine with the same peak cut-off (21–24) and that testing with GHRH alone or plus arginine be avoided, as it may yield false normal responses (21–24). The accuracy and limitations of GH stimulation tests after CRT are similar to those of the general population, but ITT seems to be the most reliable (19), while the guidelines suggest that spontaneous GH sampling should not be used as a diagnostic test. It is suggested that GH therapy (GHT) be started in those with confirmed GHD based on the safety and efficacy demonstrated in that population (18), but that it be deferred until the patient has been 1-year disease-free after the completion of therapy for malignant disease. Moreover, in childhood cancer survivors who have chronic stable disease and thus may not ever be “disease free”, it is advised to discuss with the oncologist and the family, the advisability of GHT and its timing based on a shared decision-making process. The GHT schedule and dosing is not dissimilar to that in the non-cancer population (18). A recent review and meta-analysis reported that in cancer survivors, GHT produces a higher gain in height than the untreated controls, may improve lipid profile and quality of life, and does not appear to increase the risk of diabetes or of a secondary tumor (25).

Based on the foregoing, this study was designed to evaluate how BTS patients with GHD are managed in different Italian centers and how they adhere to current guidelines. To this end, we conducted a survey among different specialists who collaborate in multidisciplinary teams to care for children BTS with GHD. A steering committee of 15 experts (11 pediatric endocrinologists, 1 neuro-oncologist and 4 neuro-radiologists) agreed to participate in the study, prepared the survey and analyzed the results of the questionnaires.

## Materials and Methods

This study was a nationwide survey of specialists’ perspectives and practices regarding GHD management in pediatric BTS in Italy. A multidisciplinary steering committee with accredited expertise in the management of GHD in pediatric BTS, including mainly pediatric endocrinologists, pediatric radiologists with neuroimaging expertise and pediatric (neuro)oncologists initially met in January 2021 to prioritize the issues to be addressed, based on their personal experience, published guidelines and more recent scientific literature.

The experts produced a survey comprised of 22 questions (**Table 1**) covering five topics: 1) organizational aspects; 2) risk factors for endocrine disorders in BTS; 3) diagnosis of GHD; 4) definition of stable disease; and 5) GHT. The survey also included two filter questions (Q1 and Q2) designed to characterize the physician’s experience in the management of pediatric BTS. All 22 questions were sent to the pediatric endocrinologists, while 16 and 9 were selected and sent to the pediatric oncologists and to radiologists, respectively, based on the field of interest.

**Table 1:** List of the 22 questions included in the survey.

Number	Question	Participants
Q1	Age of the participants	All
Q2	Number of BTS followed per year	All
Q3	In your clinical practice, is the involvement of a multidisciplinary team required when taking care of BTS patients?	All
Q4	Who is involved in the multidisciplinary team?	All
Q5	Which do you think are independent risk factors for GHD in BTS?	All
Q6	Which of these tumors do you think are more likely to cause GHD?	Endocrinologists and oncologists
Q7	Which histological types of tumor are most likely to cause GHD?	Endocrinologists and oncologists
Q8	Do you think that radiotherapy (RT) is a risk factor for GHD?	All
Q9	When would you suspect GHD in BTS?	Only for endocrinologists
Q10	Would you agree/disagree with this sentence “two GH stimulation tests are required for diagnosis of GHD”?	Only for endocrinologists
Q11	What is your clinical practice on the use of GH stimulation tests and IGF-I measurement for the diagnosis of GHD in BTS?	Only for endocrinologists
Q12	Which GH stimulation test do you use in your clinical practice for the diagnosis of GHD in an irradiated BTS?	Only for endocrinologists
Q13	How would you define a stable disease in BTS?	All
Q14	Which MRI technique/sequence would you consider to define stable disease in BTS?	All
Q15	When would you start rhGH treatment in non-craniopharyngioma survivors with established diagnosis of GHD?	Endocrinologists and oncologists
Q16	When would you start GH treatment in craniopharyngioma survivors with established diagnosis of GHD?	Endocrinologists and oncologists
Q17	Which dose of GH would you use to start therapy?	Only for endocrinologists
Q18	When would you deem it unlikely to start rhGH treatment in BTS with GHD?	Endocrinologists and oncologists
Q19	When would you stop treatment in BTS with GHD?	Endocrinologists and oncologists
Q20	Express your agreement or disagreement with the following statement: “GH therapy is safe”	Endocrinologists and oncologists
Q21	Express your agreement or disagreement with the following statement: “According to data from the literature, the risk of relapse for BTS on rhGH treatment is low”	Endocrinologists and oncologists
Q22	Would you restart GH treatment in case of newly stable disease according to imaging criteria?	Endocrinologists

All: pediatric endocrinologists, pediatric radiologists and pediatric oncologists.

Question responses were single response or multiple-choice responses depending on the nature of the question. Questions were presented as anonymous branching surveys using skip patterns to ensure customization based on the clinical profile.

The questions were administered in a web-based survey, in March 2021, to members of the steering committee and to an extended panel selected based on the following criteria: 1) management of at least 10 BTS per year; 2) tertiary care centers with specific multidisciplinary experience in the management of these children, including pediatric endocrinologists, pediatric radiologists and pediatric oncologists. The questionnaire was administered using Survey Monkey (Survey Monkey, Palo Alto, CA, USA), and the survey link was disseminated to the participants *via* a dedicated email; it was requested to avoid the use of personal information that could allow identification. All the clinicians indicated their willingness to voluntarily participate in the study. Ethics committee approval was not required for this anonymous survey.

Participants were given a response deadline of six weeks after receiving the survey, from March 12, 2021, to April 21, 2021. The survey was completed by 59 out of 96 invited specialists (61%) working overall in 37 different Centers, covering 23 cities throughout Italy. Twenty-five were pediatric endocrinologists (42%), 19 were pediatric oncologists (32%) and 15 pediatric radiologists (26%). Overall, the questionnaire was completed by at least 1 representative specialist from 32 out of the 37 centers (86.5%). The results were analyzed, discussed by the steering committee members and summarized in the results section.

## Results

### Filter Questions

Participants’ ages (Q1) ranged from 30 to over 60 years, with an average age of 54 years. Most oncologists (42%) and radiologists (47%) belonged to the 41-50 age group while most endocrinologists (44%) were more than 60 years of age.

Regarding experience in the management of pediatric BTS (Q2), 43% of the specialists involved in the survey declared that they followed more than 50 patients per year, 47% between 10 and 50 cases per year, while the remaining 10% followed about 10 BTS per year.

### Organizational Aspects

Evaluation of the BTS patient is carried out by a multidisciplinary team with specialists from the same Center in 71% (Q3), while it was supported by external specialists working as a team in 27%; no multidisciplinary evaluation was reported in 2%.

The composition of multidisciplinary teams (Q4) varied between centers. In 54% the team was composed of endocrinologist, oncologist or neurooncologist, radiologist or neuroradiologist, and neurosurgeon; in 24% additional specialists, including radiotherapists, ophthalmologists, psychologists and other health professionals were also involved. In 7% the team included endocrinologist, oncologist and neuroradiologist, and in another 7% oncologist, neurosurgeon and radiologist or neuroradiologist. Finally, 8% of participants reported working in a team of endocrinologist and oncologist.

### Risk Factors for GHD

Site of the tumor, radiotherapy and age at diagnosis were considered as independent risk (Q5) factors, respectively by 98%, 98% and 80% of the participants.

The presence of hydrocephalus was considered a risk factor by 61% (53% oncologists), while male sex and duration of follow-up were considered risk factors by 20% (11% oncologists) and 58% (47% oncologists and 84% endocrinologists), respectively (**Table 2**).

**Table 2:** Q5: Independent risk factors for GHD in BTS.

Overall	Yes	No	I don’t know
Male sex	20%	39%	41%
Age at diagnosis	80%	8%	12%
Hydrocephalus	61%	24%	15%
Follow-up duration	58%	25%	17%
Cranial radiotherapy	98%	0%	2%
Tumor localization	98%	0%	2%

**Figure 1:**
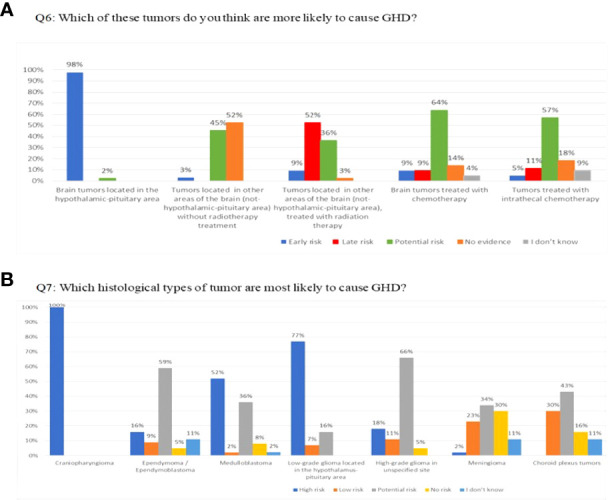
**(A)** Q6: Which of these tumors do you think are more likely to cause GHD? **(B)** Q7: Which histological types of tumor are most likely to cause GHD?

Q6 included 5 pre-defined answers (“high and early risk”; “late risk”; “possible risk”; “no risk”; “I don’t know”) for 7 conditions comprising tumors of the hypothalamic pituitary area, other cerebral tumors that did not receive CRT, other cerebral tumors that received CRT, cerebral tumors that received chemotherapy, cerebral tumors that received intrathecal chemotherapy, cerebral tumors with a history of hydrocephalus, and leukemia with no CRT (9). Almost all participants considered that tumors of the hypothalamic pituitary area carry a high and early risk of developing GHD, whereas for the majority there was a late risk or no risk for other conditions (**Figure 1A**). Fifty-three % of oncologists and 28% of endocrinologists reported that GHD can also occur after leukemia without CRT.

Q7 provided the same answers as Q6 for nine histological types of tumors. Craniopharyngiomas, germinomas of the sellar and suprasellar regions, low-grade gliomas of the hypothalamic-pituitary area, and leukemia that received CRT were considered at high risk by 100%, 93%, 77%, and 66%, respectively (**Figure 1B**). More than 50% of responders considered medulloblastomas to be at high risk.

**Figure 2:**
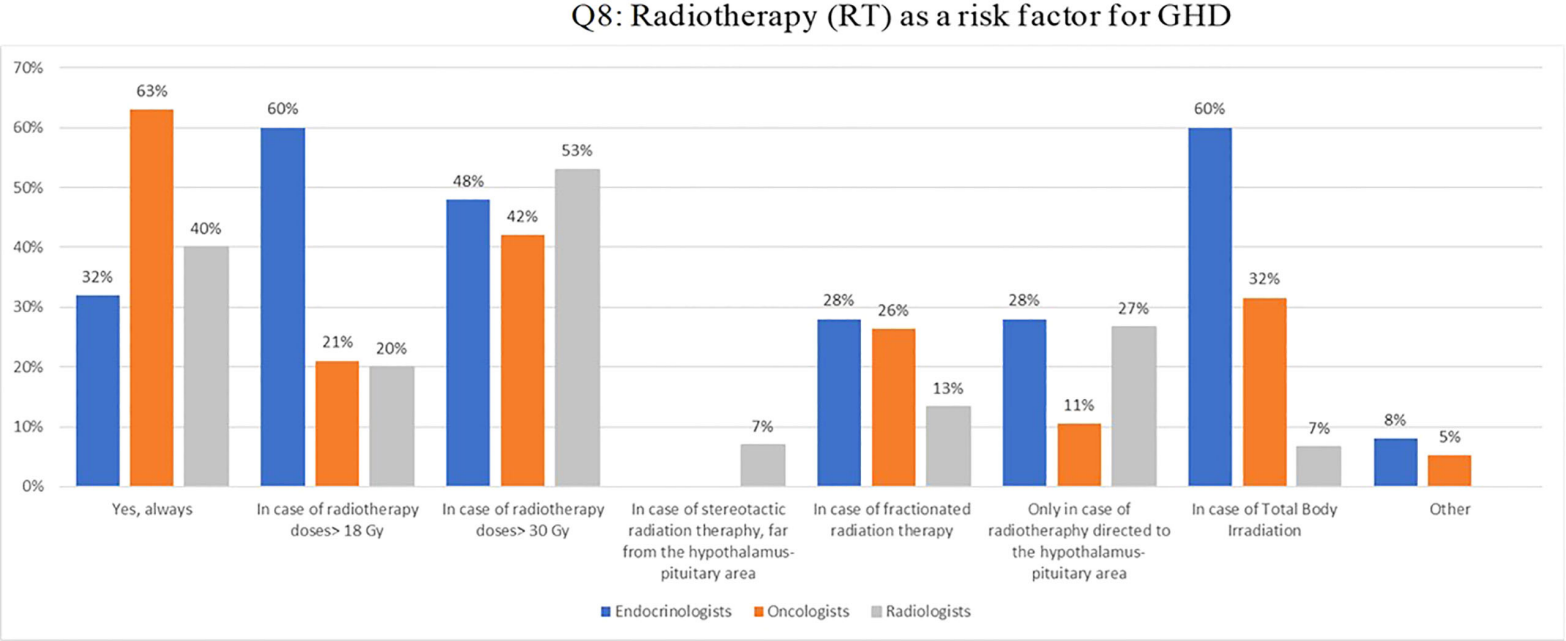
Q8: Radiotherapy (RT) as a risk factor for GHD.

Regarding radiotherapy as a risk factor for the development of GHD (Q8), 60% of endocrinologists and 21% of oncologists reported that there is some risk with doses of CRT >18 Gy, as well as 60% of endocrinologists and 32% of oncologists after TBI (**Figure 2**). Two thirds of oncologists and one third of endocrinologists believed that radiotherapy always represents a risk factor.

### Diagnosis

Questions about diagnosis of GHD in BTS were addressed only to endocrinologists.

The diagnostic suspicion of GHD (Q9) was reported above 85% for the following: signs and symptoms of hypothalamic pituitary dysfunction, growth velocity below -2 SD in one year or below -1.5 SD for two consecutive years; approximately 70% recognized the lack of growth spurt and a height loss of 0.5 SD in one year as signs of GHD.

Fifty-six percent of endocrinologists agree that two GH stimulation tests are required for a correct diagnosis of GHD (Q10), while 72% reported the need for two stimulation tests and IGF-I determination for GHD diagnosis (Q11); 20% and 8% reported the use of two stimulation tests without IGF-I measurement and one stimulation test plus IGF-I, respectively. Arginine stimulation test is used in clinical practice for the diagnosis of GHD in an irradiated BTS patient by 88%, clonidine by 48%, glucagon by 44%, insulin by 32% and GHRH plus arginine by 16% (Q12).

### Definition of Stable Disease

**Figure 3:**
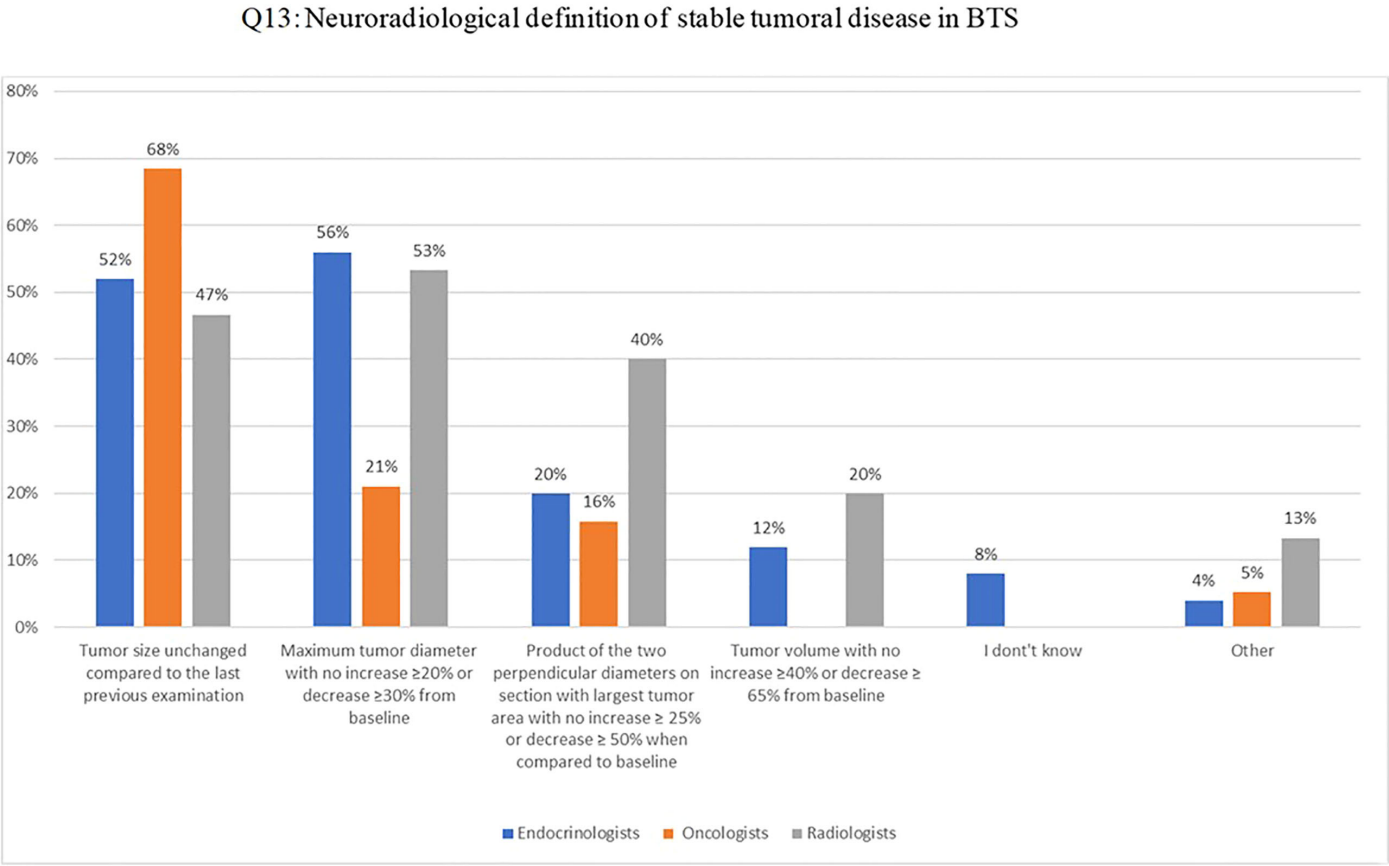
Q13: Neuroradiological definition of stable tumoral disease in BTS.

Q13 included several response options for defining stable disease in BTS, based on imaging criteria applied in neuro-oncological clinical trials: 1) one-dimensional [RECIST (Response Evaluation Criteria in Solid Tumours)]; 2) two-dimensional [RAPNO (Response Assessment in Pediatric Neuro-Oncology)], or 3) volumetric measurements of brain tumors [modified RANO (Response Assessment in Neuro-Oncology)] in comparison to baseline or best response to oncological treatment (26–28).. Fifty-two percent, 68% and 47% of endocrinologists, oncologists and radiologists, respectively, considered the disease stable when the tumor size remains unchanged based on the previous examination; the one-dimensional criteria was identified by 56%, 21% and 53%, respectively and the two-dimensional by 20%, 16% and 40%, respectively (**Figure 3**).

**Figure 4:**
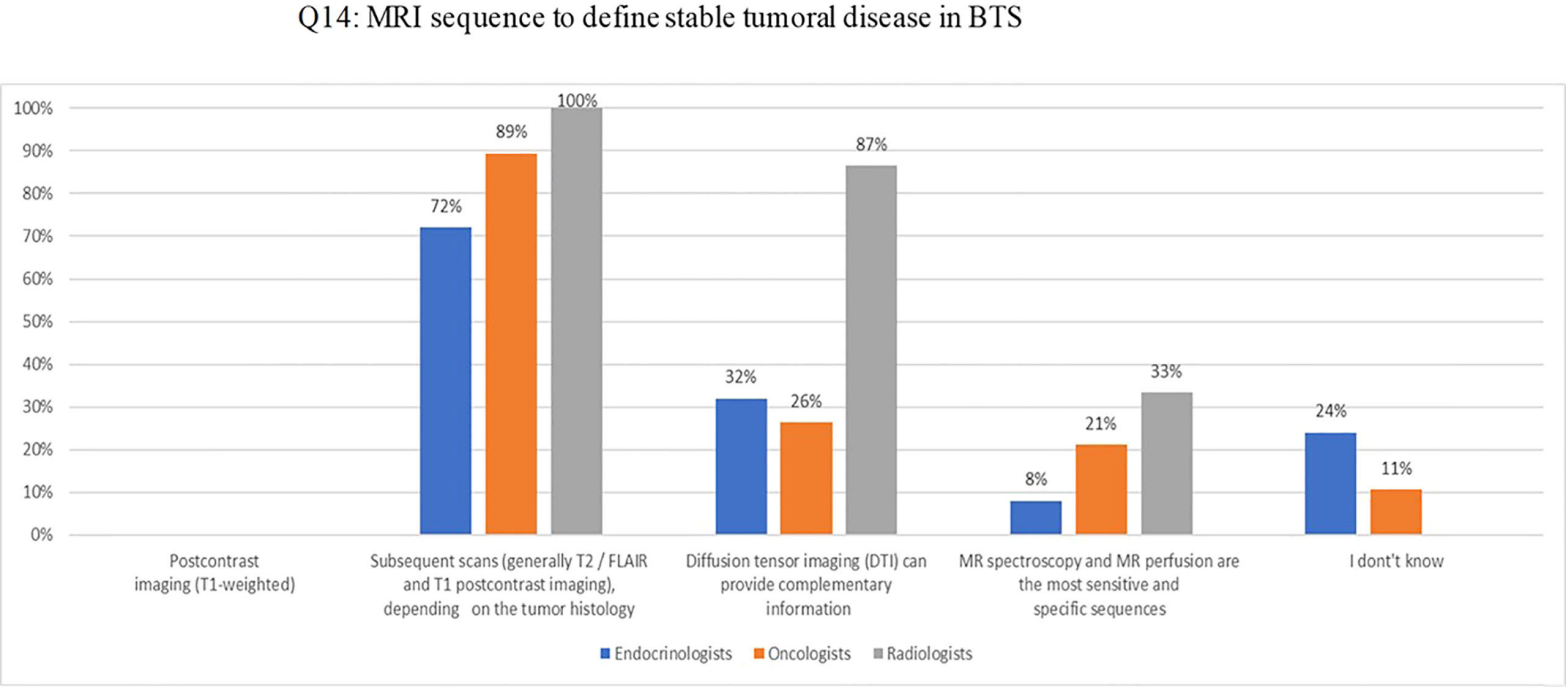
Q14: MRI sequence to define stable tumoral disease in BTS.

MRI T2/FLAIR and post-contrast T1 were considered the standard imaging sequences to define stable disease in BTS (Q14) in 100%, 89%, and 72% of the radiologists, oncologists and endocrinologists, respectively; diffusion-weighted imaging (DWI) sequence was judged useful in providing additional information by 87%, 26%, and 32%, respectively. (**Figure 4**).

### Questions on Therapy

In non-craniopharyngioma BTS with established diagnosis of GHD, 45% of endocrinologists and oncologists were confident in starting rhGH after 12 months, 25% after 24 months and 14% after 6 months of stable disease; response was reported as “other” in 16% (Q15).

In contrast, in craniopharyngioma survivors 44% of endocrinologists and 32% of oncologists would start rhGH treatment six months after complete surgical removal, while 12% and 11%, respectively, would start after 12 months. In case of partial and stable resection 40% of endocrinologists and 21% of oncologists would start treatment after 12 months, while 16% and 32% of them, respectively, would start after 24 months. None would start rhGH treatment in a non-stable condition (Q16).

Fifty-two percent of endocrinologists would start rhGH with a similar dose used in patients with hypopituitarism, 40% would titrate the GH dose according to serum IGF-I levels, 28% would start at a lower dose, and 8% would decide the dose based on the histology of the tumor (Q17 multiple choice).

Most endocrinologists and oncologists do not initiate rhGH treatment in patients with growing residual solid tumors, whether it is a craniopharyngioma (66%) or another histological type (89%) (Q18).

The majority (89%) would stop rhGH treatment in cases of critical increase of the tumor size based on the above defined neuro-imaging criteria (26–28) and in case of second tumor occurrence, including meningioma (82%). In patients who developed type 2 diabetes during rhGH, 68% of endocrinologists and 53% of oncologist would stop the treatment (Q19).

Overall, 96% agreed that rhGH treatment is safe (Q20), although endocrinologists (72%) choose more the “agree” option, while oncologists (53%) the “totally agree” option. Similarly, 86% agreed on the low risk of disease relapse in BTS induced by rhGH (Q21); however, only 40% of endocrinologists and 53% of oncologists choose the “totally agree” option. Finally, 40% of the endocrinologists would always restart rhGH treatment (Q22) in the case of newly stable disease according to the imaging criteria (26–28), regardless of histology, while 40% would restart only in patients with craniopharyngioma.

## Discussion

Growth hormone deficiency is the most common endocrinopathy in pediatric BTS and a recent study conducted in more than 3000 young adult cancer survivors reported an estimated prevalence above 40% among patients treated with CRT (6); left untreated, GHD causes decreased linear growth, adult short stature and a negative effect on cardiovascular risk and quality of life (25).

In this study, we report the results of a survey conducted in Italy among endocrinologists, (neuro)oncologists and radiologists regarding their current clinical practice in the care of pediatric BTS patients with GHD. Among the 59 specialists who participated in the survey covering 86.5% of the centers involved, the majority followed more than 50 BTS cases per year, indicating a good experience in the management of these patients. In most centers (98%), patients are followed-up by a multidisciplinary team which, reportedly, is the ideal approach to deliver the best quality of care (29). While the composition of the teams turned out to be heterogeneous, in most centers it included endocrinologists, oncologists, and radiologists.

Some discrepancies emerged in the evaluation of known risk factors for GH defect in BTS (6, 30) including time of follow-up, hydrocephalus and gender. In fact, only 58% and 61% of specialists considered the duration of follow-up and the presence of hydrocephalus as independent risk factors despite the odds ratio have been reported to be 1.14 and 12.06 in a large nationwide cohort (9). Moreover, male sex, was considered as such by only 20% of the participants despite a reported OR of 1.64 (9). Of note, 84% of the endocrinologists recognize the relevance of long-term follow-up for these patients, but only about 47% of the oncologists and 27% of the radiologists were aware of this issue. This is of particular importance since it is known that GHD can occur even several years after CRT (11–13) and that its cumulative incidence does not seem to plateau, as recently updated by international evidence-based recommendations for surveillance of hypothalamic-pituitary dysfunction (7).

Despite the strong evidence that GHD is related to tumor location and/or radiation to the hypothalamic-pituitary region, 40% of endocrinologists and 53% of oncologists considered brain tumors outside the hypothalamic-pituitary area who did not receive CRT as possible risk factors; similarly, 28% of endocrinologists and 53% of oncologists selected leukemia without irradiation as a possible risk factor, despite the fact that these patients are not at risk of developing endocrinopathies (18). However, in case of low-risk tumors, the board believes that long-term follow-up should continue.

As far as the type of tumor, there was a general agreement that craniopharyngioma and germinomas of the sellar and suprasellar region are at high and early risk of developing GHD. In addition, 52% of respondents considered medulloblastomas as an early risk factor, despite evidence that GHD occurs on average 4 years after the end of therapy and is correlated with the high dose of CRT used (12, 18).

The radiation dose was also a matter of discussion: indeed, endocrinologists were twice more confident compared to oncologists that GHD can develop after low-dose CRT (18-24 Gy) (3, 14, 31) or after TBI combined with high-dose chemotherapy for bone marrow transplant (32, 33).

Questions about diagnosis of GHD in BTS were addressed only to endocrinologists. The answers reflected a large variability of approaches, often with inadequate adherence to current international and national recommendations (18, 34). According to the most recent guidelines, GHD should be suspected in all children with known risk factors who present with short stature and/or reduced height velocity (18). There was a general agreement in recognizing signs and symptoms of hypothalamic-pituitary dysfunction and a pathological growth velocity as alert indicators for GHD; however, approximately 25% of the endocrinologists were not aware that the absence of pubertal growth spurt is also an important sign since the diagnosis is often made in this period of life.

As far as the use of GH testing for the diagnosis of GHD, 44% of the endocrinologists believe that one test is sufficient, though 80% follow the national and international recommendations that indicate, in the absence of at least 3 hormone defects, the need to perform two stimulation tests, with the exception of the GHRH plus arginine test (17, 34). Although a specific question addressing the adherence to pediatric GH cut-off levels as indicated by national guidelines was not explored (8 mcg/dl for all tests except for GHRH plus arginine, 20 mcg/dl), 80% of the participants state to follow them. Despite the majority of endocrinologists being aware that IGF-I performs poorly in the cranial irradiated cohort (13, 17) and that a single IGF-1 measurement should not be relied on, 88% of the participants included IGF-I measurements in their diagnostic GHD work-up. The need for IGF-I evaluation by the participants might reveal, on one hand, a common diagnostic approach for GHD diagnosis not differentiated for BTS, and on the other hand the existing controversy regarding the value of IGF-1 in irradiated patients. This practice is supported by a recent study showing a significant correlation between IGF-I levels and GH peak in patients who received CRT and a diagnostic value when IGF-I concentration was less than -2SDS (35).

Regarding the contribution of neuroimaging in the management of GHD in BTS, there was a general agreement on the need for brain MRI for an appropriate evaluation of these patients: MRI T2/FLAIR and post-contrast T1 were considered the standard imaging sequences and diffusion-weighted imaging (DWI) sequence was judged useful in providing additional information.

Interestingly, the definition of stable disease according to imaging criteria yielded a lively discussion, indicating divergent opinions. To date, different criteria have been proposed and applied in neuro-oncology to evaluate treatment response. Among these, RECIST ((Response Evaluation Criteria in Solid Tumours)and RAPNO (Response Assessment in Pediatric Neuro-Oncology) rely on diameter-based approaches (single largest tumor diameter and two orthogonal diameters, respectively), whereas modified RANO (Response Assessment in Neuro-Oncology) criteria include volumetric measurements (26–28). Among the response categories (i.e., partial response, stable disease or progressive disease), different cut-offs have been proposed, depending on the measurement approach. For instance, according to the RECIST method, progressive disease requires an increase of 20% or more in tumor size; this percentage is ≥ 25% with RAPNO and ≥ 40% according to the modified RANO volumetric criteria (the latter are rarely used in children). Notably, in all the different response approaches, disease is considered stable when it does not meet the criteria for progressive disease, partial response, or complete response; for example, according to the RAPNO criteria, disease is considered stable when tumor size is between an increase of < 25% or a decrease of <50%, with no evidence of new lesions (27).

All of these different criteria have been implemented in clinical trials, but the level of application and consideration in clinical practice appears to be limited. From the beginning, it is critical to define the criteria that will be used. However, 56% of the participants considered the disease stable when the tumor size remained unchanged compared to the last previous examination. This belief is not at all wrong but is incomplete, since it does not consider any of the criteria mentioned above and, most importantly, does not take into account that each MRI examination should always be analysed in comparison to the baseline or best response.

Contrast-enhanced MRI is a fundamental modality for studying the pediatric brain under a wide range of circumstances and clinical indications. In the past, the administration of contrast media was quite liberal; however, the picture changed initially with the emergence of nephrogenic systemic fibrosis, a rare cutaneous disorder that occurs in some individuals with reduced kidney function who have been exposed to intravenous gadolinium-based contrast material administrations, and more recently with concerns over gadolinium deposition in the central nervous system, bone, and other bodily organs. These problems have led to a stricter evaluation of the indications for contrast-enhanced MRI, especially in the pediatric population. However, gadolinium compounds remain a mainstay for pediatric neuro-MRI for a large number of indications, particularly tumor imaging (search for distant leptomeningeal spread, evaluation of response, surveillance imaging), evaluation of infections, and vascular imaging, among others (36, 37).

Regarding safety issues, overall, 96% agreed that rhGH treatment is safe (Q20), although endocrinologists (72%) choose more the “agree” option, while oncologists (53%) the “totally agree” option. Similarly, 86% agreed on the low risk of disease relapse in BTS induced by rhGH, however, only 40% of endocrinologists and 53% of oncologists choose the “totally agree” option. Finally, 40% of the endocrinologists would always restart rhGH treatment (Q22), regardless of histology, in case of newly stable disease according to the imaging criteria, while 40% would restart only in patients with craniopharyngioma. Interestingly, there was a high level of concerns about continuation of rhGH in case of a second tumor, including meningioma (82%). Despite the potential risk of second neoplasm associated to rhGH treatment (38), current evidence suggests the lack of risk of meningioma in patients who did not receive CRT and that GHT does not further increase the radiotherapy related-risk (39, 40).

Regarding the management of growth hormone therapy in BTS, only 45% of participants would start GH treatment in a patient with a non-craniopharyngioma brain tumor after 1 year disease-free, as suggested by the current guidelines (18), while the remaining 55% would wait 24 months or more indicating uncertainty in treating with GHT a previous tumoral patient or unawareness of recommendations. In contrast, the majority (76%) believes that rhGH treatment can be initiated after 6 months in a surgically removed craniopharyngioma. None of the participants would start treatment in a non-stable disease.

Although the decision to stop or initiate rhGH therapy should be based on well-defined and agreed-upon criteria, it is noteworthy that data on the height, metabolic and safety outcomes of rhGH therapy on stable oncological disease, as defined by one or more of the aforementioned neuroimaging criteria, are lacking. In addition, although there is general agreement that rhGH treatment is safe and does not cause residual tumor growth or recurrence, the reported answers seem to conflict with the overall “feeling” about safety. According to the current guidelines, rhGH treatment should be initiated with the same dose used in the non-cancer population and then adjusted based on serum IGF-I values (18). Indeed, there was some discordance on how to adjust rhGH since around 50% would start with a dose similar to that used in non-BTS with GHD, 40% would titrate the rhGH dose according to IGF-I concentrations, and the remaining 28% start at a lower dose.

Most endocrinologists agree not to start rhGH treatment in cases of growing residual solid tumor, whether it is a craniopharyngioma (68%) or another histological type (92%). The majority also agreed to discontinue rhGH therapy in case of unstable disease or tumor growth according to neuroimaging criteria, and about two-thirds of endocrinologists and 50% of oncologists would stop rhGH if the patient develops diabetes, though GHT does not appear to increase the risk of diabetes (25).

The strengths of this survey are that it covered over 85% of the most important Italian pediatric BTS centers and analysed in depth controversial aspects in the management of rhGH treatment in this cohort, in particular concerning the neuroradiological definition of tumor stability and the endocrinological clinical practice attitude in case of BTS patients not free from disease.

Limitations to the survey might be its reliance on answer options that could lead to some interpretation bias, i.e. the answer options “agree” or “totally agree” may be interpreted subjectively and have their own meaning to each individual respondent.

In conclusion, this survey revealed how endocrinologists, oncologists and radiologists in Italy manage pediatric cancer survivors, particularly those with brain tumors. Some areas of uncertainty as well as contrasting opinions remain on important issues, raising the question about the level of validation and appropriate adherence to important aspects of the current guidelines. These findings are relevant for specialists that take care of BTS in Italy, yet are important to highlight existing controversies worldwide, in particular in the approach of patients with residual tumor. Finally, these results emphasize the need for a closer multidisciplinary approach to the care of these patients as well as harmonization of the criteria used to define stable disease and when and how to start and stop rhGH therapy in order to improve the rhGH outcomes and the quality of life of these patients.

## Data Availability Statement

The raw data supporting the conclusions of this article will be made available by the authors, without undue reservation.

## Author Contributions

NDI, GM, AR, MM participated in the design of the study, analyzed the results of the survey, drafted and revised the manuscript. All Authors were involved in the survey design, analysis and discussion of the results, and critical review of the final manuscript. All authors approved the final manuscript as submitted and agreed to be accountable for all aspects of the work.

## Funding

This project received an unconditional grant from Merck Serono S.p.A. for medical writing and editorial assistance provided by Sanitanova S.r.l. Merck Serono S.p.A. has had no role in the survey design, collection and interpretation of data, or writing of this article. Merck Serono has performed a fairly balanced review of the publication, but the views and opinions described in the publication do not necessarily reflect those of Merck Serono.

## Conflict of Interest

The authors declare that the research was conducted in the absence of any commercial or financial relationships that could be construed as a potential conflict of interest.

## Publisher’s Note

All claims expressed in this article are solely those of the authors and do not necessarily represent those of their affiliated organizations, or those of the publisher, the editors and the reviewers. Any product that may be evaluated in this article, or claim that may be made by its manufacturer, is not guaranteed or endorsed by the publisher.
